# Patients’ Acceptance of Psoriasis Depends on Disease Severity, Itch Intensity, and the Patients’ Quality of Life: A Cross-Sectional Study

**DOI:** 10.3390/jcm13247684

**Published:** 2024-12-17

**Authors:** Beata Bień, Piotr K. Krajewski, Jacek C. Szepietowski

**Affiliations:** 1 University Centre of General Dermatology and Oncodermatology, Wroclaw Medical University, 50-556 Wroclaw, Poland; beata.bien04@gmail.com (B.B.); piotr.krajewski@umw.edu.pl (P.K.K.); 2 Department of Dermato-Venereology, 4th Military Hospital, 50-981 Wroclaw, Poland; 3 Faculty of Medicine, Wroclaw University of Science and Technology, 50-370 Wroclaw, Poland

**Keywords:** psoriasis, disease acceptance, coping strategies in psoriasis

## Abstract

**Background/Objectives:** Psoriasis is a chronic skin disorder affecting over 60 million people worldwide, with both physical and psychological impacts due to the visible lesions and associated somatic symptoms. This study aimed to assess disease acceptance among psoriasis patients and to explore its correlation with disease severity, itch intensity, and quality of life (QoL) **Methods:** The study included 166 psoriasis patients, comprising 101 men and 65 women, all with a disease duration of at least one year. Clinical and psychological aspects of psoriasis were comprehensively assessed using various standardized tools, along with a demographic questionnaire. **Results:** The study found that psoriasis patients had a mean illness acceptance score of 25.1 points, with most respondents (44%) exhibiting a moderate level of acceptance. A high level of acceptance was observed in 28% of participants, while an equal proportion of patients (28%) demonstrating low acceptance, with no significant gender-based differences. Acceptance was negatively correlated with disease severity (PASI score), quality of life impairment (DLQI), and itch intensity (NRS), indicating that lower acceptance was linked to more severe symptoms. Additionally, a slight negative correlation was observed between acceptance and disease duration, while acceptance levels were unaffected by factors such as education, relationship status, or age of disease onset. **Conclusions:** A comprehensive approach to psoriasis treatment should address not only the physical aspects but also the psychological challenges faced by patients, with a particular focus on improving disease acceptance as a coping strategy. Reducing itch intensity may contribute to better disease acceptance.

## 1. Introduction

Psoriasis is a chronic papulosquamous skin disorder categorized as immune-mediated, with dendritic cells, T-cells, and Th17 type cytokines playing pivotal roles in its pathogenesis [1]. Key factors predisposing individuals to psoriasis or contributing to disease onset include genetic components, such as the HLA-Cw6 antigen, alongside environmental influences like β-hemolytic streptococcal infections, certain medications, and psychological stress [1]. The prevalence rates of psoriasis across Europe range from 0.73% to 2.9% [2], and according to a WHO report, the disease affects more than 60 million people globally [3]. 

While skin lesions in psoriasis can develop on any part of the body, especially in severe forms, they most commonly appear on the knees and elbows, the lumbosacral region, and the scalp [4]. The most frequently reported symptoms include itching, irritation, burning/stinging, sensitivity, pain, and bleeding [5]. In addition to skin manifestations, psoriasis is associated with other prevalent conditions, such as psoriatic arthritis, metabolic syndrome, and cardiovascular disease [4]. Due to its chronic nature, psoriasis places a lasting burden on patients, who must manage ongoing treatment and diagnostic requirements, as well as the functional limitations imposed by the disease.

The impact of psoriasis becomes especially pronounced when lesions appear on highly visible areas of the body. Negative social reactions, fear of rejection, reduced interpersonal attractiveness, and the subsequent avoidance of social interactions and isolation [6] constitute a psychological threat, leading to diminished self-esteem, heightened anxiety, and depression [3,7,8,9,10]. Chronic systemic inflammation, a hallmark of psoriasis, involves elevated levels of pro-inflammatory cytokines like TNF-α, IL-6, and IL-17, which are also implicated in the pathogenesis of depression and anxiety. These cytokines can disrupt neuroendocrine signaling and alter neurotransmitter balance, contributing to mood disorders. Additionally, the hypothalamic–pituitary–adrenal (HPA) axis, often dysregulated in psoriasis, may exacerbate stress responses, further linking the skin condition to psychological distress [3,7,8,9,10]. Taking into account all the challenges associated with the disease, it is essential to implement broader therapeutic strategies that include not only the treatment of skin lesions but also psychological support aimed at enhancing the patient’s well-being. 

The literature describes various coping strategies for managing stress and the challenges associated with psoriasis, with acceptance being one such strategy [11,12]. Acceptance of illness involves recognizing the limitations imposed by the condition, understanding the available treatment options and recovery prospects, identifying accessible resources, and addressing stigma and discrimination [12,13,14]. Coping with illness is an intentional cognitive and behavioral process in which individuals implement adaptive strategies to manage the disease and sustain social functioning [14,15]. In our study, we aimed to assess the level of disease acceptance among patients with psoriasis and to investigate whether there is a correlation between the degree of acceptance and the severity of the disease, itch intensity, as well as patients’ quality of life (QoL).

## 2. Materials and Methods

### 2.1. Subjects

A cross-sectional study was conducted with an initial cohort of 200 psoriatic patients from Poland. After excluding 34 incomplete questionnaires, the final sample consisted of 166 patients, resulting in an evaluation response rate of 83%. Participation was entirely voluntary, with all individuals providing written informed consent. Anonymity was rigorously maintained, and all data were de-identified to ensure participant confidentiality. Eligibility criteria required that participants have a diagnosis of psoriasis vulgaris for at least one year. The study included adults aged 18 and older who were proficient in reading and writing in Polish. Exclusion criteria encompassed those with other chronic dermatologic conditions. At the time of assessment, all patients presented with psoriasis lesions of varying severity. The patients included in the study had not received any topical and systemic treatment for at least four weeks. The patients were biologically naïve. The severity of psoriasis was assessed by a dermatologist utilizing the Psoriasis Area and Severity Index (PASI) [16]. Demographic and clinical data were collected via a custom-designed form, followed by a series of self-administered questionnaires. The study protocol received approval from the local Ethics Committee (No. KB-234/2023).

### 2.2. Assessment of Illness Acceptance

The Acceptance of Illness Scale (AIS) [12,17] contains eight items designed to gauge how individuals cope with their illness. These items assess feelings related to worthlessness, self-reliance, limitations due to illness, and overall enjoyment of life. They also explore the respondents’ perceptions of embarrassment caused to others, dependency, and whether they feel burdensome to family and friends. Each statement is rated on a five-point scale, where 1 point represents “strongly agree”, 2 points stand for “agree”, 3 points mean “I don’t know”, 4 points indicate “disagree”, and 5 points correspond to “strongly disagree”. The total score, which can vary between 8 and 40 points, indicates the degree of illness acceptance. Results under 20 points indicate poor acceptance and adaptation to the illness, along with substantial emotional difficulties. A range of 20 to 30 points suggests moderate acceptance, whereas values above 30 points indicate high or complete acceptance.

### 2.3. Assessments of Quality of Life

The Dermatology Life Quality Index (DLQI) [18,19] was employed to assess quality of life (QoL). This validated instrument examines the impact of skin conditions on a patient’s QoL during the week prior. It includes 10 questions, with responses rated as follows: 0 points as “not at all affected” or “not relevant”, 1 point as “a little affected”, 2 points as “very much affected”, and 3 points as “affected a lot”. The highest achievable total is 30 points, with higher values indicating a greater impact on QoL.

### 2.4. Itching Assessment

The intensity of itching was measured using the Numerical Rating Scale (NRS), in which 0 indicates no itching and 10 represents the most intense itch imaginable, as experienced over the last three days [20].

### 2.5. Statistical Analysis

Statistical analyses were conducted using IBM SPSS Statistics version 26 (SPSS INC., Chicago, IL, USA). The Shapiro–Wilk test was used to assess the normality of the quantitative data. Descriptive statistics, such as the minimum, maximum, mean, and standard deviation, were calculated for the demographic and clinical characteristics.

For comparisons between groups, parametric tests, such as the T-test were conducted on normally distributed data, whereas the Mann–Whitney U test was performed on data that did not follow a normal distribution. Correlation analyses were performed with Pearson’s correlation coefficient for data with a normal distribution and Spearman’s rank correlation coefficient for data that did not follow a normal distribution. Categorical variables were analyzed using the chi-square test. When comparing more than two groups, one-way ANOVA was applied to the normally distributed data, while the Kruskal–Wallis test was employed for data that deviated from the normal distribution.

A *p*-value of ≤ 0.05 was considered statistically significant for all two-sided statistical tests.

## 3. Results

### 3.1. Demographic and Clinical Data

Among the cohort of 166 participants, 60.8% (n = 101) were males, while 39.2% (n = 65) were females, with an average age of 46 years and a standard deviation of 15.5. The educational background was primarily secondary (49.4%, n = 82) and higher education (28.9%, n = 48). Marital status showed that the majority were either married (59%, n = 98) or in informal partnerships (17.5%, n = 29), with 71.1% (n = 118) reporting having children. Clinical evaluation indicated an average Psoriasis Area Severity Index (PASI) score of 14.4 points (SD = 9.1). Participants had been managing psoriasis for an average of 19.4 years (SD = 14.8), with disease onset typically occurring at around 26.7 years of age (SD = 15.2). A significant proportion, 59% (n = 98), reported at least one psoriasis-related hospitalization. Additionally, 25.3% (n = 42) had previously received systemic therapy. Smoking rates varied notably between genders, with a higher prevalence in males (42.6%, n = 43) compared to females (23.1%, n = 15; *p* = 0.02). Table 1 provides a detailed overview of the demographic characteristics.

### 3.2. Acceptance of Illness

AIS values ranged between 8 and 40 points, with an average score of 25.1 (SD = 8.7) points. Notably, no significant differences in illness acceptance were identified based on gender. Considering the threshold values for the AIS scores, the largest percentage of participants (n = 73, 44%) demonstrated a moderate degree of illness acceptance. Meanwhile, 28% of participants (n = 47) showed a high degree of acceptance, with the same proportion of respondents (28%; n = 46) exhibiting low acceptance, with no significant gender-based differences (Table 2).

**Figure 1:**
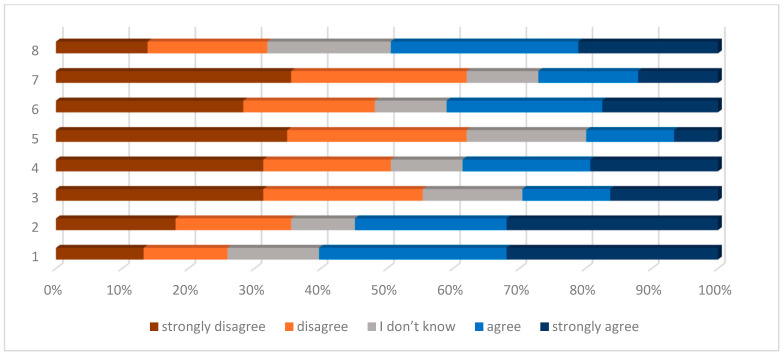
Percentage of patients’ answers on the Acceptance of Illness Scale.

An analysis of individual AIS statements revealed that adjusting to the limitations imposed by the disease (statement 1) posed the greatest challenge for respondents, with a mean score of 2.47 (SD = 1.40) points. This statement also garnered the highest levels of agreement, with 32% of respondents strongly agreeing and 28% agreeing. Additionally, the inability to engage in preferred activities (statement 2) had a mean score of 2.67 (SD = 1.52) points, with 32% strongly agreeing and 23% agreeing. Conversely, statements regarding feeling like a burden to family and friends (statement 5) and feeling dependent on others (statement 7) received the lowest levels of agreement (20% and 27%, respectively) and the highest average scores (3.7, SD = 1.26 and 3.58, SD = 1.41 points, respectively), with the highest percentage of patients disagreeing (Table 3 and Table 4, Figure 1).

**Figure 2:**
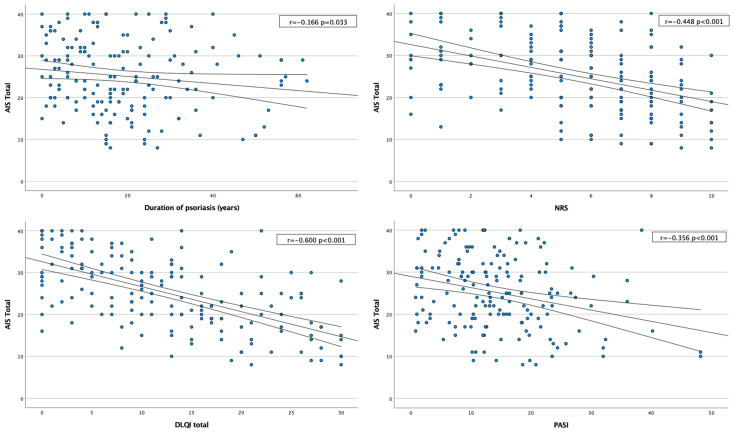
Acceptance of Illness Scale correlation with different clinical aspects.

Further analysis of the relationship between illness acceptance and psoriasis severity, as measured by the PASI score, revealed a statistically significant negative correlation (r = −0.256; *p* < 0.001). Additionally, a statistically significant negative correlation between the AIS score and the DLQI was revealed (r = −0.600, *p* < 0.001). Further analysis of different DLQI cut-offs revealed a significant difference between illness acceptance in different DLQI groups (*p* < 0.001). Furthermore, the AIS score demonstrated a strong negative correlation with itch intensity, as measured by the NRS (r = −0.448, *p* < 0.001) (Figure 2).

Interestingly, the level of acceptance of psoriasis did not significantly correlate with educational attainment, relationship status, or parenthood. However, a statistically significant negative correlation was identified between the AIS score and the duration of psoriasis (r = −0.166, *p* = 0.033). The AIS total score did not, however, show a significant correlation with the age of disease onset.

## 4. Discussion

Psoriasis is a unique chronic dermatologic condition, frequently persisting over many years. Unpredictable flare-ups, a sense of stigmatization, and the visibility of skin lesions to others create a range of challenges for patients [6]. Together with the physical symptoms, these factors significantly reduce the QoL and affect the psychological well-being of patients [8,21,22]. Literature data suggest that there is a relationship between the degree of skin illness acceptance and the intensity of psychopathological symptoms in psoriasis patients [23]. Thus, assessing and developing coping mechanisms may positively influence the patients’ psychosocial and emotional health. However, this aspect has yet to be widely applied in clinical practice and further research is needed to support its implementation.

In the study conducted by Jankowiak et al. [24], the mean AIS score for the study group was 24.3 points (SD = 6.1), with 64% of respondents demonstrating a moderate level of psoriasis acceptance. Full acceptance and complete lack of acceptance were observed in 19% and 17% of patients, respectively. Similarly, a study conducted with 366 patients in Bialystok, Poland [25], found that most patients had moderate psoriasis acceptance (46.72%), while 22.95% showed high acceptance and 30.33% low acceptance. These findings are similar to our observations. However, in another study involving 209 psoriasis patients from 12 Arabic countries [26], 38.76% reported a high level of illness acceptance, 35.9% a moderate level of acceptance, and 25.4% a low level of acceptance. Differences in illness acceptance between our country and Arabic countries may reflect various cultural, social, and religious influences on how illness is perceived and managed. However, no studies directly comparing these regions have been conducted to date.

Another interesting finding is that the level of illness acceptance observed in our group of psoriasis patients is similar to that found in conditions such as multiple sclerosis (mean 28.3, SD = 9.1 points) [27], bipolar disorder (mean 23.49 points) [13], and type 2 diabetes mellitus (mean 26.95, SD = 8.5 points) [28].

In the literature, one may find variable correlations between illness acceptance and sociodemographic factors. Some studies suggest that gender does not affect psoriasis acceptance [26,29,30], consistent with our current observations, while others indicate higher acceptance levels among men [24,25,31]. We found no studies reporting a significantly higher acceptance of psoriasis in women, which supports findings that women with skin lesions report higher levels of social anxiety, helplessness, and anxiety-depressive symptoms than men [6,32]. For women, cosmetic concerns related to skin disease may carry greater importance, influenced by societal beauty standards, which could further impact their acceptance process [6].

In our study, illness acceptance did not vary with education level, relationship status, or parenthood. However, a statistically significant negative correlation was observed between AIS scores and both disease duration and severity. As with gender, research on these sociodemographic factors and disease severity remains inconclusive [10,24,25,26,29,30], underscoring the complexity of illness acceptance and the interplay of numerous adaptive factors [6,14,29,33]. 

In a study by Moetaza [26] on psoriasis acceptance, the primary difficulties noted–similar to those in our patient group–were adjusting to the limitations imposed by the disease (statement 1: 34.4% strongly agreed, 12.9% agreed) and the inability to engage in preferred activities (statement 2: 34.4% strongly agreed, 9.1% agreed). In contrast, patients from Arabic countries strongly rejected feelings of worthlessness due to illness (statement 6: 51.7% strongly disagreed, 8.6% disagreed) and feeling like a burden to family and friends (statement 5: 50.2% strongly disagreed, 6.7% disagreed) [26].

We identified a significant correlation between illness acceptance and QoL in psoriasis patients. Greater acceptance was associated with better QoL, while lower acceptance corresponded with poorer QoL. It may seem obvious, however, the literature findings on the link between acceptance and QoL are mixed; some studies found no connection [25], while others reported significant relationships [24,26]. Psoriasis patients experiencing itching display more depressive symptoms compared to those without itching [34], and the intensity of itching significantly impacts the severity of the depressive symptoms and feelings of stigmatization [35]. Our study demonstrated that itch severity was inversely correlated with illness acceptance, meaning that higher acceptance was associated with lower itch intensity, and vice versa. To the best of our knowledge, this has never been reported until now. It has been documented that itching affects coping strategies in psoriasis patients, with severe itching being strongly linked to the following four personality traits: somatic anxiety, bitterness, mistrust, and physical aggression [36]. Furthermore, less intense and infrequent episodes of itching correlate with a stronger fighting spirit [37]. The data mentioned above may at least partly support our observations.

Beyond the development of coping mechanisms, the effective management of psoriasis requires a comprehensive, interdisciplinary approach that integrates psychological support into routine care. The study highlights the significant psychological burden of psoriasis, including its impact on self-esteem and social interactions, which underscores the importance of addressing the disease beyond its physical symptoms. Education and training for healthcare providers are pivotal in fostering a better understanding of the psychosocial challenges faced by psoriasis patients. This training should emphasize the integration of psychological and behavioral strategies into clinical practice, enabling clinicians to provide not only medical treatment but also empathetic, patient-centered care. Furthermore, a holistic management plan that includes the collaboration between dermatologists, psychologists, and social workers can help ensure that patients receive tailored support aimed at enhancing their quality of life and illness acceptance. Such a strategy reflects the necessity for a multidimensional framework in psoriasis care, which aligns with the observed correlations between illness acceptance, itch intensity, and quality of life.

## 5. Conclusions

The diagnosis of any chronic illness presents physical and psychological adaptation challenges, often requiring changes to daily routines, cessation of preferred activities, and adjustments to significant life plans [38]. A crucial mechanism for adaptation is the ability to accept both the losses associated with illness and the new responsibilities and limitations that treatment may impose [11,39].

This study has certain limitations. The cross-sectional design may not fully capture the dynamic relationship between illness acceptance and QoL, as both can change over time. Long-term studies, carried out in various geographical regions, would offer a clearer view of how acceptance evolves with disease progression. Additionally, factors such as age, gender, education, and social status can impact acceptance, but are not always considered, potentially limiting the interpretation of results.

## Figures and Tables

**Table 1:** Demographic and clinical features of the study group.

	Characteristics	Results	*p*
Sex, *n* (%):			
Females		65 (39.2)	
Males		101 (60.8)	
**Age [years] mean ± SD:**			NS
Whole group		46.0 ± 15.5	
Females		46.7 ± 16.0	
Males		45.6 ± 15.2	
**The age of psoriasis onset [years] mean ± SD:**			NS
Whole group		26.7 ± 15.2	
Females		26.0 ± 18.6	
Males		27.1 ± 12.6	
**Disease duration [years] mean ± SD:**			NS
Whole group		19.4 ± 14.8	
Females		20.7 ± 15.9	
Males		18.6 ± 14.1	
**PASI [points] mean ± SD:**			NS
Whole group		14.4 ± 9.1	
Females		12.9 ± 7.9	
Males		15.4 ± 9.7	
**NRS [points] mean ± SD:**			NS
Whole group		5.49 ± 2.79	
Females		5.55 ± 2.94	
Males		5.46 ± 2.70	
**Hospitalizations, *n* (%):**			NS
Whole group		98 (59.0)	
Females		38 (48.5)	
Males		60 (59.4)	
**Systemic treatment in the past, *n* (%):**			NS
Whole group		42 (25.3)	
Females		15 (23.1)	
Males		27 (26.7)	
**Education—primary, *n* (%):**			NS
Whole group		6 (3.6)	
Females		5 (7.7)	
Males		1 (1.0)	
**Education—vocational, *n* (%):**			NS
Whole group		30 (18.1)	
Females		8 (12.3)	
Males		22 (21.8)	
**Education—secondary, *n* (%):**			NS
Whole group		82 (49.4)	
Females		37 (56.9)	
Males		45 (44.6)	
**Education—higher, *n* (%):**			NS
Whole group		48 (28.9)	
Females		15 (23.1)	
Males		33 (32.7)	
**Marital status—single, *n* (%):**			NS
Whole group		21 (12.7)	
Females		6 (9.2)	
Males		15 (14.9)	
**Marital status—informal relationship, *n* (%):**			NS
Whole group		29 (17.5)	
Females		16 (24.6)	
Males		13 (12.9)	
**Marital status—married, *n* (%):**			NS
Whole group		98 (59.0)	
Females		36 (55.4)	
Males		62 (61.4)	
**Marital status—divorced, *n* (%):**			NS
Whole group		7 (4.2)	
Females		1 (1,5)	
Males		6 (5.9)	
**Marital status—widow/widower, *n* (%):**			NS
Whole group		11 (6.6)	
Females		6 (9.2)	
Males		5 (5.0)	
**Parenthood, *n* (%):**			NS
Whole group		118 (71.1)	
Females		49 (75.4)	
Males		69 (68.3)	
**Living alone, *n* (%):**			NS
Whole group		20 (12.0)	
Females		6 (9.2)	
Males		14 (13.9)	
**Smoker, *n* (%):**			0.02
Whole group		56 (33.7)	
Females		15 (23.1)	
Males		43 (42.6)	

*n*—number, NS—non-significant, SD—standard deviation.

**Table 2:** The degree of illness acceptance in the examined patient group.

Illness Acceptance	Whole group n, (%)	Males n, (%)	Females n, (%)	* p *
**Low**	46 (28)	26 (26)	20 (31)	NS
**Moderate**	73 (44)	45 (45)	28 (43)	NS
** High **	47 (28)	30 (30)	17 (26)	NS

n—number, NS—non-significant.

**Table 3:** Acceptance of psoriasis vulgaris as measured by the Acceptance of Illness Scale.

	Mean ± SD [Points]
**1. I have difficulty adapting to the limitations imposed by the disease.**	2.47 ± 1.40
**2. I cannot do what I like best because of my health condition.**	2.67 ± 1.52
**3. My illness makes me sometimes feel unwanted.**	3.41 ± 1.46
**4. Health problems make me more dependent on others than I would prefer.**	3.24 ± 1.54
**5. My illness makes me a burden for my family and friends.**	3.70 ± 1.26
**6. Because of my health condition, I do not feel like a truly valuable person.**	3.18 ± 1.50
**7. I will never be as self-sufficient to the extent to which I would like to be.**	3.58 ± 1.41
**8. I think people around me often feel embarrassed because of my illness.**	2.75 ± 1.35

SD—standard deviation.

**Table 4:** Distribution of responses on the Acceptance of Illness Scale.

	S-1	S-2	S-3	S-4	S-5	S-6	S-7	S-8
**Strongly agree**	32%	32%	16%	19%	7%	17%	12%	21%
**Agree**	28%	23%	13%	19%	13%	23%	15%	28%
**I don’t know**	14%	10%	15%	11%	18%	11%	11%	19%
**Disagree**	13%	17%	24%	19%	27%	20%	27%	18%
**Strongly disagree**	13%	18%	31%	31%	35%	28%	36%	14%

S—statement.

## Data Availability

Data are available upon request.
